# Risk Factors and Clinical Outcomes Associated With Acute Respiratory Distress Syndrome in Pregnant and Non-pregnant Women Diagnosed With COVID-19: A Comparative Analysis

**DOI:** 10.7759/cureus.39514

**Published:** 2023-05-26

**Authors:** Sangam Jha, Garima, Jafeesha B Singh, Shagufta Naaz

**Affiliations:** 1 Obstetrics and Gynecology, All India Institute of Medical Sciences, Patna, IND; 2 Anesthesiology, All India Institute of Medical Sciences, Patna, IND

**Keywords:** sars-cov-2, intensive care, pregnant women, covid-19, acute respiratory distress syndrome

## Abstract

Objective: We aim to compare risk factors and clinical outcomes of acute respiratory distress syndrome (ARDS) associated with coronavirus disease 2019 (COVID-19) in pregnant versus non-pregnant women of reproductive age.

Materials and methods: This retrospective study included all women (18-45 years) with ARDS and confirmed severe acute respiratory syndrome coronavirus 2 (SARS-CoV-2) infection admitted during the study period from May 2020 to July 2021. Pregnant women were considered as case and non-pregnant women as control. Primary outcomes included ventilatory support, the need for high-flow nasal oxygenation (HFNO), severe ARDS, and death. Secondary outcomes included intensive care unit (ICU) admission, length of hospital stay, and requirement of oxygen at discharge.

Results: We included 59 women diagnosed with ARDS and confirmed SARS-CoV-2 infection, of them 12 were pregnant and 47 were non-pregnant. The non-pregnant women were significantly older than pregnant women (28.7±5 versus 35.5±8.2, p=0.008). Presenting symptoms were comparable among the groups. Diabetes was significantly higher in the non-pregnant group (8.3% versus 31.9%, p<0.02). Pregnant women had a significantly higher range of D-dimer (5.8±7.2 versus 1.8±1.9, p<0.01) and interleukin-6 (IL-6) (212.0±300.8 versus 49.7±57.7, p<0.011) and lower platelet count (129.4±120.1 versus 197.6±92.9, p<0.05) compared to non-pregnant women. Pregnant women were more likely to experience primary outcomes including the need for HFNO (33% versus 8.5%, odds ratio (OR): 5.3, p<0.02) and death (50% versus 31.9%, OR: 2.1, p<0.04) compared to non-pregnant women.

Conclusion: Pregnant women with severe COVID-19 and ARDS were at an increased risk for experiencing ICU admission, intubation, and mechanical ventilation compared to age-matched non-pregnant women, although comorbidities such as diabetes were higher among the non-pregnant cohort. These findings suggest that pregnancy itself is a potential risk factor for complications and morbidities among women with severe COVID-19.

## Introduction

The coronavirus disease 2019 (COVID-19) is an acute infectious pneumonia caused by a severe acute respiratory syndrome coronavirus 2 (SARS-CoV-2) previously unknown to humans. Since the outbreak in early December 2019 in Wuhan, China, it has become a global pandemic. The course of the disease is highly unpredictable and varies from asymptomatic viremia to fatal respiratory distress syndrome. Acute hypoxemic respiratory failure in the presence of diffuse lung opacities with impairment in gas exchange is the hallmark presentation of acute respiratory distress syndrome (ARDS). It has been reported that 67% of COVID-19 patients with severe illness have developed ARDS, which is the main cause of death [1]. The impact of COVID-19 on pregnant women is relatively unknown. Given the adaptive immunological alteration and physiological changes, pregnancy is a potentially vulnerable state for COVID-19. In addition, these changes to the immune and respiratory systems increase vulnerability to severe infection and hypoxic compromise. There is limited information regarding the outcome of severe COVID-19 in pregnant women. Recently, few studies have reported a higher risk of intensive care unit (ICU) admission and mechanical ventilation during pregnancy [2,3], whereas studies from Europe and North America concluded that pregnant women were at no increased risk of severe COVID‐19 or death [4-6]. So, predicting various risk factors and clinical features with which pregnant women are more likely to develop ARDS compared to non-pregnant women and thus face a greater risk of complications including death is particularly important in a novel and accelerating outbreak. This study aimed to compare risk factors and clinical outcomes of ARDS associated with COVID-19 in pregnant versus non-pregnant women of reproductive age.

## Materials and methods

This was a retrospective observational study conducted at a single tertiary care center from May 2020 to July 2021. This study was approved by the institutional ethics committee (AIIMS/PAT/IEC/730). All women aged 18-45 years with confirmed severe SARS-CoV-2 infection and who met the inclusion criteria for ARDS were included in the study. Those women having asymptomatic, mild/moderate COVID-19 infection or negative RT-PCR results were excluded from the study. Data pertaining to demographic characteristics, clinical course, laboratory and radiological parameters, and obstetrical and clinical outcomes were obtained from the patients’ record sheets.

A test result was considered positive if the quantitative reverse transcription PCR (qRT-PCR) analysis of the nasopharyngeal swab was positive for SARS-CoV-2. The Berlin definition was used to clinically define ARDS, which includes (1) onset within seven days of a known insult, most commonly pneumonia or sepsis, (2) presence of diffuse lung opacities in chest imaging, and (3) evidence of hypoxemia as defined by a ratio of partial pressure of arterial oxygen to fraction of inspired oxygen (PaO2/FiO2) ≤ 300 mmHg, at a minimum positive end-expiratory pressure (PEEP) of 5 cmH20 [7]. Respiratory function was assessed using estimated partial pressure of arterial oxygen/fraction of inspired oxygen (ePaO2/FiO2) ratio, which had been calculated by pulse oximetry saturation/fraction of inspired oxygen (SpO2/FiO2) ratio (SpO2/FiO2 = 64 + 0·84 × PaO2/FiO2) [8]. Cases were defined as pregnant women with ARDS associated with confirmed SARS-CoV-2 infection. Controls were defined as non-pregnant females of the reproductive age group (18-45 years) with confirmed SARS-CoV-2 infection and were diagnosed with ARDS.

Primary outcomes included ventilatory support, the need for high-flow nasal oxygenation (HFNO), severe ARDS, and death. Secondary outcomes included ICU admission, length of hospital stay, and requirement of oxygen at discharge.

We also explored obstetrical data relating to maternal and fetal outcomes. Maternal and fetal complications were defined as per guidelines from the Federation of Obstetric and Gynaecological Societies of India.

Statistical analysis

Quantitative data have been shown as the mean±standard deviation (SD) or the median with interquartile range (IQR). Qualitative variables were expressed as absolute and relative frequencies. Categorical variables were compared using the χ2 test or Fisher’s exact test. Student’s t-test or Mann-Whitney U test was applied for continuous variables, as appropriate. A p-value of <0.05 was considered statistically significant. All statistical analyses were done using MedCalc Statistical software version 19.2.6 (MedCalc Software bvba, Ostend, Belgium) (http://www.medcalc.org (2020)).

## Results

A total of 669 women of reproductive age with confirmed SARS-CoV-2 infection were admitted during the study period. Of the 669 women, 59 met the criteria for ARDS. Of note, 12 women were pregnant, considered as case, and 47 were non-pregnant, taken as control.

The demographic and clinical characteristics of the studied population are outlined in Table 1. Non-pregnant women were significantly older than pregnant women (35.5±8.2 versus 28.7±5, p=0.008). Presenting symptoms were comparable among both groups. Diabetes was significantly higher in the non-pregnant group (31.9% versus 8.3%, p<0.02). Heart disease (8.3% versus 4.2%), hypertension (41.6% versus 23.4%), and liver disease (16.6% versus 4.2%) were more commonly seen in the pregnant group compared to the non-pregnant group, but the difference did not reach statistical significance.

**Table 1:** Demographic and clinical characteristics of the studied population ePaO2/FiO2 ratio: estimated partial pressure of arterial oxygen/fraction of inspired oxygen

Characteristics	Pregnant (number (%)/mean±SD) (n=12)	Non-pregnant (number (%)/mean±SD) (n=47)	p-value
Age (years)	28.7±5	35.5±8.2	0.008
Symptoms			
Fever (>38.4°C)	10 (83%)	37 (78.7%)	0.96
Difficulty in breathing	11 (91.6%)	42 (89.3%)	0.94
Myalgia	3 (25%)	3 (6.3%)	0.17
Gastrointestinal	2 (16.6%)	8 (17%)	0.68
Others	3 (25%)	2 (4.2%)	0.08
Preexisting comorbidity			
Diabetes mellitus	1 (8.3%)	15 (31.9%)	0.02
Heart disease	1 (8.3%)	2 (4.2%)	0.57
Hypertension	5 (41.6%)	11 (23.4%)	0.21
Renal disease	2 (16.6%)	8 (17%)	0.68
Liver disorder	2 (16.6%)	2 (4.2%)	0.37
Anemia (Hb < 10.5 gm%)	7 (58.3%)	22 (46.8%)	0.69
ePaO_2_/FiO_2_ ratio	203.4±154.9	170.5±134.3	0.46

Table 2 compares the laboratory parameters of both groups. Pregnant women had a significantly higher range of D-dimer (mg/L) (5.8±7.2 versus 1.8±1.9, p<0.01) and interleukin-6 (IL-6) (pg/mL) (212.0±300.8 versus 49.7±57.7, p<0.011) compared to non-pregnant women. Platelet count was lower among the pregnant cohort compared to the non-pregnant cohort (129.4±120.1 versus 197.6±92.9, p<0.05).

**Table 2:** Biochemical markers among patients with severe COVID-19 COVID-19: coronavirus disease 2019, CRP: C-reactive protein, ALT: alanine transaminase, AST: aspartate transaminase, IL-6: interleukin-6, LDH: lactate dehydrogenase, NLR: neutrophil/lymphocyte ratio, SD: standard deviation

Biochemical markers	Pregnant (mean±SD)	Non-pregnant (mean±SD)	p-value
CRP (mg/dL)	75.2±58.7	94.4±69.7	0.46
ALT (IU/L)	102.8±156.7	93.2±205	0.89
AST (IU/L)	84.4±72.6	70.3±77.1	0.60
D-dimer (mg/L)	5.8±7.2	1.8±1.9	0.01
Hemoglobin (mg/dL)	8.6±2.8	13.2±1.8	0.44
IL-6 (pg/mL)	212.0±300.8	49.7±57.7	0.011
LDH (IU/L)	1102.4±442.8	1377.2±2290	0.72
NLR (%)	10.7±9.5	12.6±9.1	0.54
Platelet count (lac/mL)	129.4±120.1	197.6±92.9	0.05
Procalcitonin (ng/mL)	1.0551±0.9	4.2630±2.7	0.51

Pregnant women were more likely to experience primary outcomes including the need for HFNO (33% versus 8.5%, odds ratio (OR): 5.3, p<0.02) and death (50% versus 31.9%, OR: 2.1, p<0.04) compared to non-pregnant women. The need for mechanical ventilation (66.6% versus 48.9%, p<0.43) and tracheostomy (16.6% versus 4.2%, p<0.12) was greater among the pregnant group than that of the non-pregnant group; however, the difference did not reach statistical significance. Pregnant women were more likely to experience secondary outcomes including ICU admission and discharge with oxygen requirement, but none were statistically significant. Table 3 summarizes the outcome of patients among pregnant and non-pregnant groups.

**Table 3:** Outcome of patients among pregnant and non-pregnant groups n: total number of pregnant women, N: pregnant woman with a specific outcome, SD: standard deviation, CI: confidence interval, ICU: intensive care unit, HFNO: high-flow nasal oxygenation, ARDS: acute respiratory distress syndrome

Outcome	Pregnant (N/n (%)/mean±SD)	Non-pregnant (N/n (%)/mean±SD)	Odds ratio (95%CI)	p-value
Severe ARDS (PaO2/FiO2: 200-300 mmHg)	4/12 (33.3%)	11/47 (23.4%)	1.6 (0.41-6.48)	0.47
Moderate ARDS (PaO2/FiO2: 100-200 mmHg)	1/12 (8.3%)	2/47 (4.2%)	2 (0.16-24.65)	0.57
Mild ARDS (PaO2/FiO2: <100 mmHg)	7/12 (58.3%)	34/47 (72.3%)	0.53 (0.14-1.99)	0.35
Ventilatory support	8/12 (66.6%)	23/47 (48.9%)	2.7 (0.6-11.8)	0.43
HFNO	4/12 (33%)	4/47 (8.5%)	5.3 (1.1-26)	0.02
Death	6/12 (50%)	15/47 (31.9%)	2.1 (0.5-7.7)	0.04
ICU admission	10/12 (83.3%)	33/47 (70%)	2.1 (0.4-10.9)	0.36
Tracheostomy	2/12 (16.6%)	2/47 (4.2%)		0.12
Cardiac arrest	0	1		
Respiratory failure	0	1		
Discharged on room air	5/12 (41.6%)	27/47 (57.4%)	0.5 (0.14-1.9)	0.32
Discharged with home oxygen requirement	1/12 (8.3%)	2/47 (4.2%)	2 (0.16-24.6)	0.36
Duration of hospital stay (in days)	13.2±9.5	17.3±15.2		0.38

Table 4 briefly outlines the maternal and perinatal outcomes of pregnant women with ARDS. Seven patients had a cesarean delivery, whereas three patients were delivered vaginally. Three babies were born prematurely, and fetal demise occurred in three women. Two pregnant women were discharged antenatally.

**Table 4:** Maternal and neonatal outcomes of pregnant women with severe COVID-19 COVID-19: coronavirus disease 2019, HELLP: hemolysis elevated liver enzyme low platelet, PIH: pregnancy-induced hypertension, IHCP: intrahepatic cholestasis of jaundice, PPH: postpartum hemorrhage, RDS: respiratory distress syndrome

Outcome	Number
Gestational age at delivery	
<28 weeks	3
28-34 weeks	4
34-37 weeks	2
>37 weeks	3
Mode of delivery	
Cesarean section	7
Vaginal delivery	3
Indication of delivery	
Maternal	5
Fetal	1
Obstetrical	2
Obstetric complications	
Twin pregnancy	2
Eclampsia	1
HELLP syndrome	1
PIH	3
IHCP	2
Fetal demise	3
Postpartum complication	
PPH	1
Puerperal sepsis	1
Neonatal complications	
RDS	3
Prematurity	3

## Discussion

In this comparative analysis, we observed that pregnant women had suffered a severe form of ARDS (33.3% versus 23.4%) and experienced more severe outcomes such as death and requirement of HFNO compared to non-pregnant women. They were also more likely to experience ICU admission and mechanical ventilation, which are proxy markers of severe disease, compared to non-pregnant women.

The clinical presentation of COVID-19 infection in pregnant women was similar to those reported by non-pregnant women. Fever and breathing difficulty were the commonest symptoms among both groups. This is consistent with the observation made by Chen et al. [9]. Previous literature has demonstrated that the presence of comorbidities such as diabetes, heart disease, cancer, and hypertension increases the severity of COVID-19 disease [10,11]. In the present study, we observed an increased incidence of diabetes among the non-pregnant cohort compared to the pregnant cohort (8.3% versus 31.9%, p<0.02). However, pregnant women experienced more severe outcomes such as death, mechanical ventilation, or ICU requirement, which indicates that pregnancy itself increases the risk of severity for COVID-19 disease. A recent review of nine studies comprising 591,058 women (28,797 pregnant and 562,261 non-pregnant) by Khan et al. [12] also reported that COVID-19-infected pregnant women had a greater requirement of ICU admission and invasive mechanical ventilation compared to non-pregnant women, despite having a higher frequency of risk factors among non-pregnant women.

Laboratory parameters that characterize SARS-CoV-2 infection were analyzed. D-dimer (5.8±7.2 versus 1.8±1.9, p<0.01) and IL-6 (212.0±300.8 versus 49.7±57.7, p<0.011) were significantly higher and platelets (129.4±120.1 versus 197.6±92.9, p<0.05) were lower in the pregnant group compared to the non-pregnant group. Physiological changes in biochemical indices during pregnancy including thrombocytopenia, increased D-dimer, CRP, and lymphocytosis might explain the differences observed among the groups. IL-6 is an indigenously expressed cytokine during pregnancy and helps maintain cellular homeostasis throughout gestation in fetal membrane cells. Increased production of IL-6 from fetomaternal tissue in response to infection is a nonspecific innate response and is an indicator of adverse pregnancy outcomes [13]. This might explain the higher concentration of IL-6 in the pregnant cohort. No other significant differences in laboratory indices had been observed between the groups in this study. These findings are consistent with the observation made by Mohr-Sasson et al. [14], wherein they reported no significant difference in laboratory indices among pregnant and non-pregnant cohorts. Zha et al. [15] also reported mild differences in the laboratory characteristics of pregnant women compared to non-pregnant women; however, they suggested that these differences were due to physiological and immunological changes during pregnancy rather than a disease per se.

We have found a higher risk of severe ARDS (33.3% versus 23.4%), ICU admission (83.3% versus 70%), and need for mechanical ventilation (66.6% versus 48.9%) among the pregnant group compared to the non-pregnant group. However, the difference did not reach statistical significance. This may be explained by the small sample size. The requirement for high-flow nasal oxygen was significantly higher (33% versus 8.5%, OR: 5.3, p<0.02) among the pregnant group, and they also experienced higher mortality (50% versus 31.9%, OR: 2.1, p<0.04) compared to non-pregnant women. Both groups received similar management, including antiviral drugs, steroids, low-molecular-weight heparin, and prone positioning. Our findings are in agreement with the previous multicentric study from the United States that included 38 pregnant women with severe and critical COVID-19 disease, wherein they reported higher composite morbidity (high-flow nasal cannula supplementation, noninvasive positive pressure ventilation, intubation or mechanical ventilation, extracorporeal membrane oxygenation, and death) of 34.2% versus 14.9% (p<0.03) among the pregnant cohort compared to the non-pregnant cohort [16]. Collin et al. [3] also reported that pregnant women with confirmed SARS-CoV-2 infection were 2.59 times more likely to require intensive care compared to non-pregnant women. The largest case narrative on COVID-19 pneumonia in pregnancy, from Spain, showed that pregnant females had a 61.5% chance of developing pneumonia with a severe course in more than half of cases as compared to the general population between age groups 30 and 40 years [17]. They also suggested the presence of bilateral lung infiltrates and elevated serum CRP at admission as a risk factor for developing severe COVID-19 pneumonia. A systematic review by Allotey et al. [18] comprising 11,432 pregnant and recently postpartum women had described an increased risk for ICU admission and mechanical ventilation among those with confirmed SARS-CoV-2 infection.

Most of the pregnant women who delivered during the hospital course had a cesarean delivery (70%, 7/10). Three babies were born prematurely, one of which was from twin gestation at <32 weeks and two were between 34 and 37 weeks gestational age. Three fetal demise had occurred in this series, one from twin gestation, one at <28 weeks gestation, and one at term with eclampsia. Maternal and fetal outcomes in this study are in agreement with previous literature, which demonstrated a positive association between severe COVID-19 disease and adverse pregnancy outcomes [19].

This study has a few limitations. We could not match the cases with control according to age, body mass index (BMI), and disease severity. Therefore, we compared pregnant women with women of reproductive age group (18-45 years). The risk of inherent bias exists as most of the data were collected retrospectively. The threshold for intensive care admission and initiating oxygenation had been low for pregnant women for maintaining an adequate uteroplacental system. This study has many strengths as well. We have included 59 subjects with confirmed SARS-CoV-2 infection along with ARDS and compared pregnant women who were specifically admitted for the disease to a non-pregnant cohort. Management protocols were similar among both groups. We had observed a greater risk of primary outcomes such as mechanical ventilation and death among pregnant women, which could not be subjected to bias based on pregnancy status.

## Conclusions

Pregnant women with severe COVID-19 diagnosed with ARDS are at an increased risk of morbidity compared to age-matched non-pregnant women of reproductive age. We did not notice significant differences in laboratory parameters between the groups. Although comorbidities such as diabetes were higher among the non-pregnant cohort, pregnant women remained at increased risk of experiencing ICU admission, intubation, mechanical ventilation, and tracheostomy. The length of hospital stay was similar between our cohorts. These findings suggest that pregnancy itself is a potential risk factor for complications and morbidities among women with severe COVID-19. Our study suggests that COVID-19 increases the risk of adverse pregnancy outcomes too. This information has important implications while counseling pregnant women diagnosed at the primary stage of the disease on the potential for advancing to a severe stage.

## References

[1] Yang X, Yu Y, Xu J (2020). Clinical course and outcomes of critically ill patients with SARS-CoV-2 pneumonia in Wuhan, China: a single-centered, retrospective, observational study. Lancet Respir Med.

[2] Ellington S, Strid P, Tong VT (2020). Characteristics of women of reproductive age with laboratory-confirmed SARS-CoV-2 infection by pregnancy status - United States, January 22-June 7, 2020. MMWR Morb Mortal Wkly Rep.

[3] Collin J, Byström E, Carnahan A, Ahrne M (2020). Pregnant and postpartum women with SARS-CoV-2 infection in intensive care in Sweden. Acta Obstet Gynecol Scand.

[4] Gajbhiye RK, Modi DN, Mahale SD (2020). Pregnancy outcomes, Newborn complications and Maternal-Fetal Transmission of SARS-CoV-2 in women with COVID-19: a systematic review. medRxiv.

[5] Breslin N, Baptiste C, Gyamfi-Bannerman C (2020). Coronavirus disease 2019 infection among asymptomatic and symptomatic pregnant women: two weeks of confirmed presentations to an affiliated pair of New York City hospitals. Am J Obstet Gynecol MFM.

[6] Knight M, Bunch K, Vousden N (2020). Characteristics and outcomes of pregnant women admitted to hospital with confirmed SARS-CoV-2 infection in UK: national population based cohort study. BMJ.

[7] Ferguson ND, Fan E, Camporota L (2012). The Berlin definition of ARDS: an expanded rationale, justification, and supplementary material. Intensive Care Med.

[8] Rice TW, Wheeler AP, Bernard GR, Hayden DL, Schoenfeld DA, Ware LB (2007). Comparison of the SpO2/FIO2 ratio and the PaO2/FIO2 ratio in patients with acute lung injury or ARDS. Chest.

[9] Chen L, Li Q, Zheng D (2020). Clinical characteristics of pregnant women with Covid-19 in Wuhan, China. N Engl J Med.

[10] Hasan J, Anam AM, Huq SM, Rabbani R (2021). Impact of comorbidities on clinical outcome of patients with COVID-19: evidence from a single-center in Bangladesh. Health Scope.

[11] Ge E, Li Y, Wu S, Candido E, Wei X (2021). Association of pre-existing comorbidities with mortality and disease severity among 167,500 individuals with COVID-19 in Canada: a population-based cohort study. PLoS One.

[12] Khan DS, Pirzada AN, Ali A, Salam RA, Das JK, Lassi ZS (2021). The differences in clinical presentation, management, and prognosis of laboratory-confirmed COVID-19 between pregnant and non-pregnant women: a systematic review and meta-analysis. Int J Environ Res Public Health.

[13] Omere C, Richardson L, Saade GR, Bonney EA, Kechichian T, Menon R (2020). Interleukin (IL)-6: a friend or foe of pregnancy and parturition? Evidence from functional studies in fetal membrane cells. Front Physiol.

[14] Mohr-Sasson A, Chayo J, Bart Y, Meyer R, Sivan E, Mazaki-Tovi S, Yinon Y (2020). Laboratory characteristics of pregnant compared to non-pregnant women infected with SARS-CoV-2. Arch Gynecol Obstet.

[15] Zha Y, Chen G, Gong X (2021). Coronavirus disease 2019 in pregnant and non-pregnant women: a retrospective study. Chin Med J (Engl).

[16] DeBolt CA, Bianco A, Limaye MA (2021). Pregnant women with severe or critical coronavirus disease 2019 have increased composite morbidity compared with nonpregnant matched controls. Am J Obstet Gynecol.

[17] San-Juan R, Barbero P, Fernández-Ruiz M (2020). Incidence and clinical profiles of COVID-19 pneumonia in pregnant women: a single-centre cohort study from Spain. EClinicalMedicine.

[18] Allotey J, Stallings E, Bonet M (2020). Clinical manifestations, risk factors, and maternal and perinatal outcomes of coronavirus disease 2019 in pregnancy: living systematic review and meta-analysis. BMJ.

[19] Villar J, Ariff S, Gunier RB (2021). Maternal and neonatal morbidity and mortality among pregnant women with and without COVID-19 infection: the InterCovid multinational cohort study. JAMA Pediatr.

